# Alcohol and Stress Together Can Lead to Long‐Term, Dementia‐Like Deleterious Effects in the Brain

**DOI:** 10.1111/acer.70308

**Published:** 2026-05-01

**Authors:** Thatiane De Oliveira Sergio, Frederic W. Hopf

**Affiliations:** ^1^ Department of Psychiatry Indiana University School of Medicine (IUSM) Indianapolis Indiana USA; ^2^ Stark Neurosciences Research Institute IUSM Indianapolis Indiana USA

Alcohol drinking can potentiate stress responses, while stress can impair behavioral control and elevate alcohol intake. In humans, chronic excessive alcohol use produces many brain changes including loss of gray and white matter and disruptions in synaptic functions, neuronal excitability, and signaling pathways. Stress can similarly alter brain function, and stress and alcohol can both accelerate development of dementia‐like behaviors, such as impaired cognition and emotional regulation. Importantly, alcohol and stress likely interact in a self‐reinforcing cycle, where alcohol weakens stress coping and increases anxiety, while stress impairs behavioral control and drives further alcohol drinking (Sinha 2022). A recent paper in *ACER*, from Revka et al. (2026), investigates whether combined stress and high alcohol exposure could produce effects on drinking and cognitive function even after 3 months of abstinence from alcohol. In parallel, the authors examined molecular changes in the Locus Coeruleus (LC), the brain's primary source of norepinephrine, which supports executive function, attention, memory, and stress regulation, and is also implicated in dementia. For example, Alzheimer's disease (AD) involves degeneration of LC neurons and disrupted norepinephrine signaling, contributing to cognitive decline (Chen et al. 2022).

Thus, deficits in LC function following combined stress and alcohol could provide critical mechanistic insight into how stress+alcohol can be a risk factor for developing dementia. To investigate these possibilities, the authors exposed C57/BL6 mice to voluntary alcohol drinking 1 h per day for 3 weeks, in order to establish a baseline of volitional intake. Mice then underwent four cycles of alcohol vapor (CIE) and forced swim stress (FSS) before alcohol drinking. Specifically, in each cycle, mice had four nights of 16 h alcohol vapor, and then 1 week of volitional alcohol drinking with daily forced swim stress before each drinking session. This CIE/FSS method has been used by several groups, and alcohol drinking levels are reliably increased in sessions with forced swim just before drinking. Revka and colleagues replicated this finding, with elevated post‐FSS drinking in CIE/FSS mice compared to controls with air/no‐FSS when tested in the final drinking session of the four cycles.

We note that CIE/FSS have forced swim stress before their drinking, while controls do not. Thus, higher drinking levels in CIE/FSS could be a function of the previous stress/high alcohol exposure and also the presence of FSS before the drinking session. In practice, this may mean that CIE/FSS conditions lead to elevated drinking when stress is present, rather than enhancement of alcohol intake, per se. Even so, it is likely that human drinkers have multiple stressors in their lives, including those caused by or related to their alcohol intake (Larimer et al. 1999; Weckesser et al. 2025). However, some acute stressors decrease or do not change drinking rather than increase it (Lopez et al. 2016; Weckesser et al. 2025). Thus, repeated experience of high alcohol drinking in the context of high stress may be particularly effective at anchoring neuroadaptations that elevate future drinking and intake‐related harms.

After four cycles of CIE/FSS, mice underwent 3 months of abstinence and were tested at 11–12 months of age (midlife). First, CIE/FSS mice underwent one more cycle of CIE, then FSS and drinking. CIE/FSS mice continued to drink more than controls, indicating persistent, long‐term increases in alcohol intake. Drinking levels were stable over time: mice classified as high or low drinkers before abstinence remained so afterward, suggesting no incubation effect but rather sustained elevations in stress‐related drinking.

After testing drinking post abstinence, cognitive function was then assessed using the Barnes maze, which measures spatial learning, memory, and cognitive flexibility. Briefly, the task involves a circular platform with 20 evenly spaced holes along the perimeter and elevated ~70 cm above the ground. The room has bright overhead lighting and a white noise generator, which encourages escape behavior. The mice then need to use spatial cues to learn which of the 20 holes is the target hole which allows escape, and memory is tested 48 h after acquisition. One week after testing, the escape location was changed to assess reversal learning, providing a measure of cognitive flexibility.

Interestingly, CIE/FSS mice show equivalent spatial learning to controls, suggesting that there are no deficits in the ability to learn where the target hole was, or in the latency to approach the target hole. In contrast, CIE/FSS mice show a disrupted ability to learn the reversed condition. After reversal, CIE/FSS mice spend less time in the new target location, making significantly more errors and trending to take longer to locate the new escape hole. Taken together, CIE/FSS mice clearly show deficits in cognitive flexibility after 3 months of abstinence and ~2.5 weeks of post‐abstinence drinking.

After Barnes maze testing, brain tissue was collected for immunohistochemical analysis of the LC. CIE/FSS mice show elevated levels of HNE, a marker of lipid peroxidation, indicating increased oxidative stress in LC neurons compared to controls. In contrast, there are no significant differences in the number of caspase‐6‐positive cells, suggesting no detectable increase in apoptosis at this stage. These findings indicate that earlier life CIE/FSS exposure produces persistent oxidative damage in the LC that remains evident at midlife and may precede cell loss at later ages. The authors also then examined mRNA expression of the α2 adrenergic receptor (α2AR) in LC cells, which act as auto receptors to reduce norepinephrine release. CIE/FSS mice have significantly lower α2AR mRNA levels in LC cells, ~20% lower compared to controls. Thus, LC cells likely have greater norepinephrine release in CIE/FSS exposed mice compared to controls at midlife.

The authors suggest that elevated oxidative stress and elevated norepinephrine release might contribute to the impaired reversal performance in CIE/FSS mice. Norepinephrine function is known to be U‐shaped, such that moderate norepinephrine can increase cognitive function, while higher norepinephrine during stress can impair cognitive function (Arnsten 2015; Snyder et al. 2012; Valentino and Van Bockstaele 2008). Since spatial learning per se was not disrupted, this might suggest that the elevated challenge of reversing a response makes reversal susceptible to the disruptive impact of higher norepinephrine levels. Alternately, oxidative damage might be the primary disruption, leading to impaired release of norepinephrine, and α2AR decreases could be a compensatory change to enhance norepinephrine release. Light‐based norepinephrine sensors, and direct patch clamp of LC neuron firing activity, might shed valuable light on the functional state of LC cells in CIE/FSS mice.

It is also interesting that α2AR activators such as guanfacine can reduce human alcohol drinking and cocaine use (Fox et al. 2023). Reducing norepinephrine release can reduce craving for addictive substances and decrease the related negative mood and anxiety. One possibility is that human addicts have significant stress‐related high intake during at least some portions of their life, leading to elevated norepinephrine levels which drive seeking and intake. However, it remains somewhat controversial how much anxiety/stress actually promote intake in human addicts, since many heavy drinkers report that the rewarding aspects of alcohol drive their continued intake (Grodin et al. 2019; King et al. 2025). However, a subset of human drinkers report that anxiety and relief promote their continued intake (Grodin et al. 2019), and this is more prevalent in women (Erol and Karpyak 2015). If stress‐related drinking accelerates development of dementia‐like states, then anxiety drinkers might be at greater risk for this condition.

We note that targeting α2ARs is perhaps a less promising strategy for the treatment of dementia. Several dementia and other age‐related conditions are associated with disrupted LC and norepinephrine function, but this may occur in stages. Moderate LC dysfunction could lead to elevated norepinephrine levels and symptoms such as emotion dysregulation and aggression. Earlier studies showed that AR blockers can reduce aggression in AD patients (Peskind et al. 2005), and brexpiprazole (Rexulti) is now marketed to reduce this agitation, although this compound impacts multiple neuromodulators. However, at more advanced AD stages, where LC degeneration is more progressed, the brain may have limited norepinephrine reserves, and α2AR activators (which would reduce norepinephrine levels) would be contraindicated. Instead, atomoxetine, which inhibits norepinephrine and dopamine transporters and thus elevates norepinephrine levels, could be tested for increasing cognitive function and/or reducing AD progression (Demsey et al. 2025).

These points underscore the complexity and need for care and caution when targeting the norepinephrine system, especially in the elderly. The norepinephrine system regulates a number of cognitive and emotional functions, and other signaling such as neuro‐inflammation. In addition, as noted above, norepinephrine effects are often U‐shaped, where moderate norepinephrine increases can enhance cognitive flexibility while higher levels mediate stress impairments in cognition. Thus, the norepinephrine system remains of great interest, including for clinical applications, and it would be of value for further work to define the conditions where adrenergic receptor and related modulators are helpful versus harmful.

One consideration in the work of Revka and colleagues is that mice underwent 3 months of abstinence but then received an additional session of CIE and FSS‐related drinking. This provides a limitation to the possibility that reversal deficits and LC changes were long‐lasting across abstinence. For example, mice with 3 months of abstinence but did not receive a final CIE FSS testing might not show reversal deficits or LC changes. In this view, cognitive and cellular differences would result from the final CIE/FSS cycle after the abstinence period. Indeed, the authors note that others have found reversal learning deficits during early withdrawal from alcohol or stress exposure, although humans with AUD but with protracted abstinence also have reversal deficits, supporting their findings. Thus, to ascertain that CIE/FSS led to long‐lasting changes, it would have been optimal to test cognitive and LC function without the final drinking test after abstinence. However, it is recognized that these are laborious studies, and adding another cohort of CIE/FSS versus control mice adds significant additional work. In addition, aging studies of LC suggest that cellular changes as observed here typically occur at older ages. Nonetheless, changes in the norepinephrine/AR system in mice after alcohol exposure are observed earlier in life (Varodayan et al. 2025).

Another consideration is that there are notable sex differences in norepinephrine function, including in relation to alcohol (Valentino and Van Bockstaele 2008; Varodayan et al. 2025). Here, the sexes were combined, and thus, even with strong overall effects of CIE/FSS, it would be valuable to determine whether sex differences might be observed in the long‐term impact of stress/alcohol exposure.

Finally, it would be interesting to determine if CIE/FSS treatments lead to more macroscopic changes in the brain, such as decreases in gray matter and white matter volume. Such changes are observed in humans with AUD, and one major obstacle is the lack of rodent models which recapitulate alcohol‐related brain degeneration seen in humans. With the profound changes in LC oxidative stress at the midlife time point, it may be that the CIE/FSS model could produce broader histological changes as the mouse ages even further, mirroring an increasingly dementia‐like profile. Thus, the authors present interesting and suggestive studies that point to the next round of experiments to even better understand the long‐term impact of stress‐related high alcohol exposure.

## Funding

This work was supported by the National Institute on Alcohol Abuse and Alcoholism, AA030710.

## Conflicts of Interest

The authors declare no conflicts of interest.

## Data Availability

Data sharing not applicable to this article as no datasets were generated or analysed during the current study.
